# Incidence and impact of respiratory tract infections on outcomes in patients with status epilepticus: A 20‐year longitudinal observation

**DOI:** 10.1002/epi.70458

**Published:** 2026-09-17

**Authors:** Sebastian Berger, Sira M. Baumann, Urs Fisch, Lisa Hert, Pascale Grzonka, Sabina Hunziker, Stephan Rüegg, Sarah Tschudin‐Sutter, Raoul Sutter

**Affiliations:** ^1^ Clinic for Intensive Care Medicine, Department of Acute Medicine University Hospital Basel Basel Switzerland; ^2^ Interdepartmental Division of Critical Care Medicine University of Toronto Toronto Ontario Canada; ^3^ Department of Neurology University Hospital Basel Basel Switzerland; ^4^ Medical Communication and Psychosomatic Medicine University Hospital Basel Basel Switzerland; ^5^ Medical Faculty, University of Basel Basel Switzerland; ^6^ Division of Infectious Diseases, Department of Acute Medicine University Hospital Basel Basel Switzerland

**Keywords:** patient outcomes, respiratory tract infection, status epilepticus, ventilator‐associated pneumonia

## Abstract

**Objective:**

This study was undertaken to identify the incidence of respiratory tract infections (RTIs) in a large cohort of adult patients with status epilepticus (SE) and to characterize their associations with clinical features and outcome determinants.

**Methods:**

This was a retrospective observational study in the intensive care unit (ICU) at the University Hospital of Basel, Switzerland from 2005–2024. All adult patients treated for SE at the ICU were included. We recorded the incidence of RTI diagnosed during or 48 h after SE termination and clinical characteristics. Outcome characteristics potentially complicated by RTI (i.e., SE duration, hospital stay, survival with return to premorbid neurological function at hospital discharge, and mortality) were secondary objectives.

**Results:**

Among 818 patients (median age = 68 years, 49% female), 34.2% required mechanical ventilation. RTIs were diagnosed in 12.0% (98/818), rising to 34.3% (96/280) among ventilated patients, with 21.4% (21/98) meeting ventilator‐associated pneumonia criteria. Characteristics associated with subsequent RTI included nonconvulsive SE (NCSE) with coma, unconsciousness at onset, and early intubation (odds ratio [OR] = 1.8–2.6); 53.4% (31/58) of patients with all three features developed RTIs. Patients with RTI had higher maximal median markers of inflammation (C‐reactive protein with RTI 81 mg/L vs. without RTI 23 mg/L, *p* < .001; leukocytes with RTI 11 × 10^9^/L vs. leukocytes without RTI 10 × 10^9^/L, *p* = .002). After adjusting for age, etiology, and SE severity, RTI remained associated with an 8.44‐day increase in SE duration (95% confidence interval [CI] = 5.31–11.56), a 54% reduced likelihood of full neurologic recovery (OR = .46; 95% CI = .26–.81), and more than double the risk of in‐hospital mortality (OR = 2.12, 95% CI = 1.13–4.00).

**Significance:**

RTIs affect more than one third of mechanically ventilated patients with SE and approximately every second intubated patient with NCSE and coma. RTIs were associated with coma, early intubation, higher mortality, and longer SE duration. Prospective studies are needed to clarify the direction of these associations and whether preventing RTIs improves outcomes.

**Key points**

- RTIs occurred in 12% of all adult patients with SE and in 34.3% among those requiring mechanical ventilation.
- A total of 53.4% of patients with early intubation, unconsciousness at onset, or NCSE with coma developed an RTI.
- Diagnosis of RTI remains associated with longer durations of SE, lower rates of full neurological recovery, and higher in‐hospital mortality even after adjusting for age, seizure etiology, and SE severity.
- Diagnosis of RTI is associated with increased peak levels of blood‐based biomarkers of systemic inflammation. We theorize that these infections cause systemic inflammation that sustains or exacerbates seizures, providing a rationale for the hypothesis that prophylactic antibiotics may improve outcomes in these SE patient subgroups.

## 1 INTRODUCTION

Respiratory tract infections (RTIs), including ventilator‐associated pneumonia (VAP), occur frequently in neurocritical care populations, possibly facilitated by impaired airway protection and aspiration.^1^, ^2^ Due to their relationship with poor outcomes, prevention strategies are under investigation. Two recent meta‐analyses of antibiotic prophylaxis in neurocritical care patients found that selective antibiotic prophylaxis reduces the risk of VAP and decreases the length of stay in patients with acute brain injury.^3^, ^4^

Data regarding VAP and RTI‐specific outcomes in status epilepticus (SE) are limited to small cohort studies reporting RTI incidence rates of 16%–29% with VAP rates of 6%–8% without analyses of clinical associations.^5^, ^6^, ^7^, ^8^

In a study of 160 adults, peri‐ictal infections significantly increased mortality and the risk of refractory SE.^6^ Respiratory infections occurring within 72 h of onset were particularly linked to longer SE duration, extended intensive care unit (ICU) stays, and increased mortality. Subsequent analysis of a larger cohort of 311 patients showed that ICU care for >24 h was associated with an increased rate of complications and prolonged ventilation was associated with increased mortality and poor neurological recovery.^7^ These results are in line with another study revealing an independent association of prolonged postictal mechanical ventilation with death.^9^

Although these data underscore that RTI and duration of mechanical ventilation are associated with morbidity and mortality, further confirmation by larger studies and investigations regarding the risk profile are lacking.

The primary objectives of this study were to determine the incidence of RTIs in a large cohort of adult patients with SE and to identify characteristics at SE onset associated with subsequent RTI and outcome characteristics potentially complicated by RTI.

## 2 MATERIALS AND METHODS

### 2.1 Approvals and patient consents

The study protocol was approved by the ethics committee of Northwestern and Central Switzerland (EKNZ 2019‐00693). In accordance with the Declaration of Helsinki, the requirement for informed consent was waived. This study follows the Strengthening the Reporting of Observational Studies in Epidemiology (STROBE) guidelines.^10^

### 2.2 Data collection

This Swiss longitudinal retrospective observational study was conducted at the University Hospital of Basel, an academic tertiary care center. Data were derived from the STEPS (Swiss Status Epilepticus Population Study) registry (ClinicalTrials.gov: NCT04204863, date of registry: December 17, 2019). This cohort included all consecutive adult patients (aged ≥18 years) presenting with SE between January 2005 and January 2024. Datasets were extracted from institutional electronic health records and integrated into a secure REDCap (Research Electronic Data Capture) database to ensure data fidelity and privacy.^11^

SE was categorized according to the dominant seizure type at SE onset as convulsive for cases with convulsive seizures >5 min, whereas other SE types (including myoclonic forms and nonconvulsive types [nonconvulsive SE (NCSE)] with or without coma) were diagnosed if respective seizures lasted for ≥10 min electrographically, as defined by the Salzburg criteria. The diagnosis of SE was extended to include instances where recurrent seizures occupied more than 20% of a 1‐h continuous electroencephalographic (cEEG) recording period.^12^, ^13^

The duration of SE was defined as the interval between the initial clinical and/or electrographic seizure onset and the EEG‐confirmed termination of seizures. Because in some cases no cEEG but intermittent spot EEGs were performed for a minimum of 20 min every 12 h, the calculated SE duration represents an approximation with a potential margin of error of ≤12 h.

The temporal onset of SE was determined based on phenotypic presentation, with motor‐prominent SE identified through clinical manifestation and NCSE characterized by electrographic detection. For patients requiring anesthesia, SE termination was defined as a seizure‐free EEG pattern following anesthetic titration and withdrawal. Treatment refractoriness was classified according to the Neurocritical Care Society guidelines, necessitated by failure of both initial benzodiazepines and subsequent nonbenzodiazepine antiseizure medications.^14^

Semiologic classification of SE was aligned with International League Against Epilepsy (ILAE) standards,^12^ distinguishing between SE with prominent motor features, comprising convulsive and myoclonic subtypes and NCSE, further stratified by the presence or absence of coma and its focal or generalized nature.^12^, ^15^ Presumed underlying etiologies were categorized into acute symptomatic causes, remote symptomatic origins, and progressive neurologic disorders. However, the primary classification was dictated by the treating physician's assessment of the dominant ILAE‐defining factor. Consistent with established exclusion criteria, patients with posthypoxic SE were excluded. Subsequently, etiology of SE was further categorized as potential nonfatal and fatal etiologies as defined elsewhere.^16^, ^17^

The duration of SE and the Status Epilepticus Severity Score (STESS; scaled from 0 to 6^18^, ^19^) were used to quantify severity of SE. The Charlson Comorbidity Index (scaled from 0 to 37^20^) was used to quantify risk related to non‐seizure‐related comorbid conditions. Systematic assessment of clinical complications focused on the incidence of severe arterial hypotension requiring continuous vasopressor support, multiorgan failure, and the emergence of secondary infectious sequelae.

### 2.3 Antiseizure treatment

SE was managed according to Neurocritical Care Society and American Epilepsy Society guidelines, under the continuous supervision of neurologists and neurointensivists.^14^, ^21^ Initial intervention consisted of intravenous benzodiazepine administration. Upon the failure of first‐line agents, second‐line therapy was escalated. Cases progressing to RSE necessitated a multimodal pharmacological approach, incorporating nonsedating antiseizure medications, alongside the initiation of continuous intravenous anesthetic infusions. These anesthetic agents were titrated to achieve either the complete cessation of electrographic seizures or a burst–suppression pattern on EEG, according to individual clinical judgment.^22^, ^23^, ^24^ In instances of breakthrough seizure activity or recurrence, barbiturates were typically utilized as a salvage therapy to establish and maintain deeper burst–suppression.

### 2.4 Assessment of infectious complications

Infectious complications were retrospectively assessed by comprehensive review of medical and microbiological records as previously described.^6^, ^25^ An additional chart review was performed for this study during June 2026 adding information on timing of antibiotic initiation, maximum levels of leukocytes and C‐reactive protein (CRP) levels during SE, and type and duration of antibiotic therapy. General infections were identified by integrating clinical examination, radiologic imaging, and laboratory results according to Centers for Disease Control and Prevention criteria.^26^

The diagnosis of RTI was established following the guidelines of the Centers for Disease Control and Prevention and required a combination of imaging, clinical, and laboratory criteria: at least one of the following: (1) fever of >38°C or (2) leukocyte count of <4000/μL or >12 000/μL; and at least two of the following: (1) new onset of purulent sputum or change in character of sputum, or increased respiratory secretions, or increased suctioning requirements; (2) dyspnea or tachypnea, or new onset or worsening cough; (3) rales or bronchial breath sounds; or (4) worsening gas exchange and consistent radiographic findings (new and persistent or progressive and persistent infiltrates, consolidations, or cavitations).^26^

Given the high sensitivity and low specificity of clinical suspicion for VAP, diagnostic confirmation adhered to the stringent guidelines by the Centers for Disease Control and Prevention.^26^ The diagnosis of VAP, a subgroup of patients with RTI, was specifically reserved for pulmonary infections developing at least 48 h following endotracheal intubation. Microbiological confirmation required pathogen isolation from lower respiratory tract specimens obtained through transtracheal aspiration, bronchial brushing, or tissue biopsy or via positive blood cultures. The temporal scope of this infectious surveillance spanned the entire duration of the post‐SE hospitalization, including a retrospective window of 14 days preceding the onset of the index neurological event.

### 2.5 Treatment of RTI and VAP

During the study period, in‐house guidelines adapted from international guidelines based on the local epidemiology were followed.

For community‐acquired pneumonia,^27^ amoxicillin was recommended for mild cases, amoxicillin/clavulanate (or ceftriaxone) in initial combination with clarithromycin for potential atypical pneumonia, and broad‐spectrum beta‐lactams combined with a macrolide or fluoroquinolone was recommended for severe cases at risk for *Pseudomonas aeruginosa* infection. Recommended treatment duration was 5–7 days.^28^

For hospital‐acquired pneumonia and VAP,^29^, ^30^ ceftriaxone or amoxicillin/clavulanate was recommended for patients without risk factors for multidrug‐resistant pathogens, whereas piperacillin/tazobactam or cefepime was recommended to cover for patients with risk factors for more resistant pathogens. Anti‐methicillin‐resistant *Staphylococcus aureus* coverage with Vancomycin was not recommended empirically due to low domestic rates. Antibiotic treatment of hospital‐acquired pneumonia was recommended for 5–7 days^31^ with prolonged treatment of 10–14 days only in patients presenting with complications, such as bacteriemia or empyema, or in patients with delayed clinical improvement.

### 2.6 Primary and secondary outcomes

The incidence of RTI originating during SE and clinical characteristics preceding SE were defined as primary outcomes. The incidence of RTI originating during SE was defined as including any RTI diagnosed between SE onset and 48 h after SE termination. In line with the time‐critical definition of VAP^26^ regarding in‐ and extubation, any RTI and VAP diagnosed up to 48 h after the termination of SE was defined as having originated during SE.

“Intubation at SE onset” was defined as endotracheal intubation performed immediately at the time of SE diagnosis/onset as part of the initial management, whereas “mechanical ventilation” included all patients who required invasive mechanical ventilation at any time during their ICU stay, irrespective of the timing of intubation.

Outcome characteristics potentially complicated by RTI (i.e., SE duration, hospital stay, survival with return to premorbid neurological function at hospital discharge, and mortality) were secondary objectives. Survival with return to premorbid neurological function was defined as either the complete resolution of neurological deficits or the return to the patient's baseline functional status documented prior to the SE event.

### 2.7 Statistics

Descriptive statistics for categorical variables were reported as absolute counts and corresponding percentages, whereas continuous metrics were summarized via medians and interquartile ranges (IQRs).

Patients were categorized into two groups based on whether they were diagnosed with RTI during SE. Comparisons between these two groups were performed with chi‐squared or Fisher exact tests, depending on the requirements of the underlying data frequencies for proportions, and Mann–Whitney *U*‐tests for continuous variables. The level of significance for univariable analyses was set at *p* ≤ .002 after Bonferroni correction for multiple comparisons.

Multivariable logistic regression, including stepwise forward and backward, as well as Akaike information criterion, was performed to identify associations between variables at SE onset and the emergence of RTI independent of potential confounders. To further identify associations between RTI and secondary outcomes, uni‐ and multivariable linear and logistic regression models were calculated, including established outcome predictors of SE, as previously outlined.^32^, ^33^ Tests for adequate model fit (as indicated by insignificant *p*‐values) were performed using the Hosmer–Lemeshow goodness of fit tests. To assess whether associations between RTI and secondary outcomes differ after excluding patients with VAP, sensitivity analysis was performed. The two‐sided level of significance was set at a *p*‐value of <.05 for the logistic regression models. Statistical analysis was performed with Stata 16.1 (StataCorp).

### 2.8 Use of artificial intelligence‐assisted technologies

Generative artificial intelligence (AI; Gemini 3 Flash, Google) assisted in linguistic optimization, grammatical organization, and drafting the discussion. It was also used to paraphrase and condense manuscript sections. All AI‐generated content underwent manual revision to ensure scholarly accuracy and consistency with the primary data.

## 3 RESULTS

A total of 818 adult patients with SE were included in this longitudinal study, with 34.2% (280/818) requiring mechanical ventilation. Additional demographic, clinical, laboratory, and treatment characteristics are presented in Table 1. The median age of the cohort was 68 years, and 49.0% (401/818) were female. The most frequent leading SE etiologies grouped by ILAE criteria were acute symptomatic seizures (39.4%, 322/818) and epilepsy without additional triggers (19.9%, 163/818), and 32.3% (264/818) of cases were attributed to presumed fatal etiologies. Regarding the clinical presentation and SE semiology, 40.6% (332/818) of patients presented with prominent motor symptoms, whereas 59.4% (486/818) presented with NCSE. Median SE severity, as quantified by the STESS, was 3 (IQR = 2–4).

**TABLE 1:** Demographics, clinical characteristics, treatment, and outcomes (*N* = 818).

Demographics and clinical characteristics	Total cohort	
	*n*/median	%/IQR
Demographics		
Age, years, median, IQR	68	55–78
Female, *n*, %	401	49.0
**Clinical characteristics**		
Leading SE etiology grouped according to the ILAE, *n*, %^15^		
Acute symptomatic seizures	322	39.4
Remote symptomatic unprovoked seizures	139	17.0
Progressive CNS disorders	106	13.0
Epilepsy without additional triggers (with or without underlying electroclinical syndrome)	163	19.9
Cryptogenic	87	10.6
Presumed fatal etiologies, *n*, %^16^, ^17^	264	32.3
Main presumed SE etiologies (non‐mutually exclusive), *n*, %		
Known epilepsy	192	23.5
Brain tumors	103	12.6
Remote ischemic stroke	89	10.9
Intracranial hemorrhage	83	10.2
Drug withdrawal	49	6.0
Traumatic brain injury	41	5.0
Infectious (meningo‐)encephalitis	40	4.9
Neurodegenerative	37	4.5
Acute ischemic stroke	34	4.2
Metabolic/electrolyte derangement	28	3.4
Autoimmune encephalitis	28	3.4
Septic encephalopathy	11	1.3
Intoxication	9	1.1
Posterior reversible encephalopathy syndrome	7	.9
Drug effects	1	.1
SE type, *n*, %		
SE with prominent motor symptoms	332	40.6
SE with motor symptoms (convulsive)	225	27.5
SE with motor symptoms (myoclonic)	107	13.1
SE without prominent motor symptoms	486	59.4
NCSE with coma	189	23.1
NCSE without coma	297	36.3
Generalized NCSE without coma (i.e., atypical absence status)	6	.7
Focal NCSE without coma (conscious)	52	6.4
Focal NCSE without coma (with altered consciousness)	239	29.2
Illness severity, median, IQR		
STESS	3	2–4
CCI	4	2–5
Treatment characteristics of SE		
ICU treatment, *n*, %	631	77.1
Intubation at SE onset, *n*, %	97	11.9
Mechanical ventilation, *n*, %	280	34.2
Duration of mechanical ventilation, days, median, IQR	6	2–14
Number of nonanesthetic antiseizure drugs, median, IQR	3	2–4
Patients with continuous anesthetic drugs, *n*, %	240	29.3
Treatment characteristics of infections (in 142 patients with infections during SE)		
Antibiotic treatment, *n*, % of 142	130	91.5
Time from SE onset to antibiotic treatment, days, median, IQR	0	0–0
Duration of antibiotic treatment, median, IQR	7	6–11
Type of antibiotics, *n*, % of 142		
Co‐amoxicillin	47	33.1
Ceftriaxone	43	30.3
Piperacillin‐tazobactam	21	14.9
Others	19	13.4
No antibiotic treatment	8	5.6
Antivirals	4	2.8
Systemic inflammation characteristics during SE		
Maximal C‐reactive protein serum levels, mg/L, median, IQR	27.1	6.6–77.3
Maximal leukocyte counts, ×10^9^/L, median, IQR	10.4	7.7–13.4
Noninfectious complications during SE, *n*, %		
Arterial hypotension requiring vasopressors	113	13.8
Organ failure	81	9.9
Primary outcomes, *n*, %		
Infections	142	17.4
Respiratory tract infection	98	12.0
Ventilator‐associated pneumonia	21	2.6
Secondary outcomes		
In‐hospital stay, days, median, IQR	13	6–24
ICU stay, days, median, IQR	4	2–11
SE duration, days, median, IQR	1	.5–2
Persistent seizure termination, *n*, %	778	95.1
Survival with return to premorbid neurologic function at discharge, *n*, %	338	41.3
In‐hospital death, *n*, %	78	9.5

Abbreviations: CCI, Charlson Comorbidity Index (range = 0–37)^20^; CNS, central nervous system; ICU, intensive care unit; ILAE, International League Against Epilepsy^12^, ^15^; IQR, interquartile range; NCSE, nonconvulsive SE; SE, status epilepticus; STESS, Status Epilepticus Severity Score (range = 0–6).^18^, ^19^.

### 3.1 Primary outcomes: Incidence and clinical associations of RTI

Respiratory tract infections were diagnosed in 12% (98/818) of patients (Figure 1). Of mechanically ventilated patients, 34.3% (96/280) had RTI. Only .4% (2/538) of patients without mechanical ventilation developed RTI. Among RTIs, 21.4% (21/98) fulfilled the diagnostic criteria for VAP. The median time from SE onset to RTI diagnosis was 1 day (IQR = 0–2), whereas VAP was diagnosed at a median of 3 days (IQR = 2–5). Univariable comparisons of patients with and without diagnosed RTI regarding demographics and clinical characteristics at SE onset are outlined in Table 2. After correction for multiple comparisons with a level of significance set at *p* ≤ .002, these analyses identified several clinical characteristics at SE onset as being significantly associated with the emergence of RTI, including NCSE with coma (46.9% [46/98] vs. 19.9% [143/720] in those without RTI, *p* < .001), having a higher median STESS (3 [IQR = 2–4] vs. 3 [IQR 2–5], *p* = .001), being unconscious at SE onset (62.2% [61/98] vs. 31.7% [228/720], *p* < .001), and requiring intubation at SE onset (31.6% [31/98] vs. 9.2% [66/720], *p* < .001). Uni‐ and multivariable logistic regression analyses to identify associations at SE onset with the emergence of RTI are presented in Table 3. In all multivariable logistic regression models, including forward and backward selection and Akaike information criterion, NCSE with coma, unconsciousness at SE onset, and intubation at SE onset remained associated with the diagnosis of RTI, with high odds ratios (ORs) for RTI of between 1.8 and 2.6. Of note, in the 58 patients with all these three characteristics (presenting with NCSE with coma, unconsciousness at SE onset, and intubated at SE onset), 53.4% (31/58) had RTI.

**FIGURE 1:**
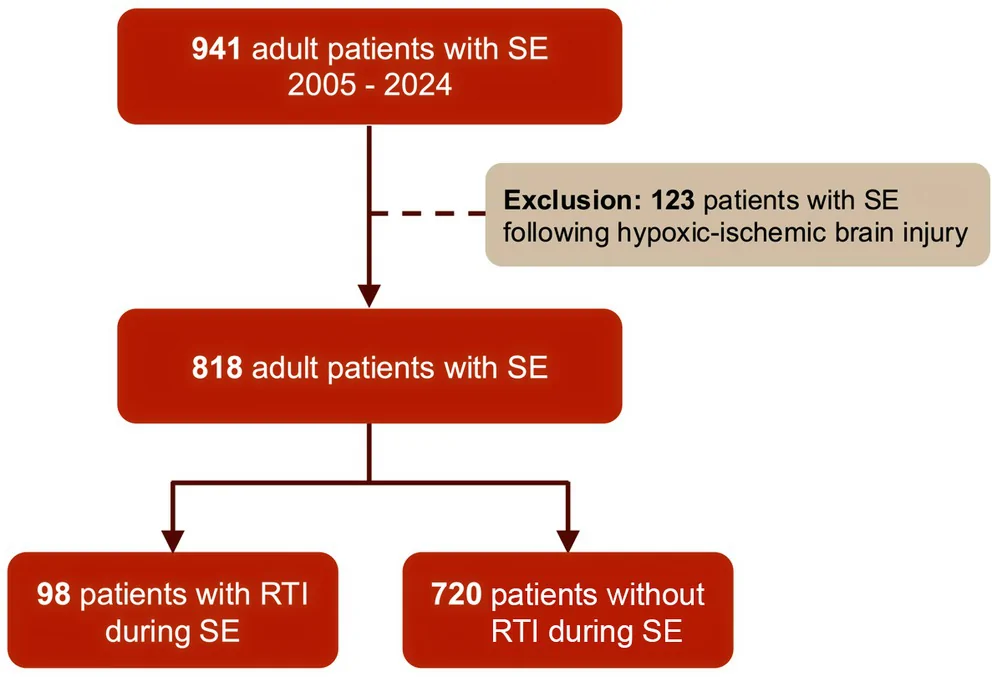
Flowchart. RTI, respiratory tract infection; SE, status epilepticus.

**TABLE 2:** Univariable comparisons of demographics and clinical characteristics at SE onset between patients with and without respiratory tract infections (*n* = 818).

Demographics and clinical characteristics	Patients without RTI during SE, *n* = 720		Patients with RTI during SE, *n* = 98		*p* ^a^
	*n*/median	%/IQR	*n*/median	%/IQR	
Demographics					
Age, years, median, IQR	69	56–78	64	52–73	.013
Female, *n*, %	354	49.2	47	48.0	.822
Clinical characteristics					
Leading SE etiology grouped according to the ILAE, *n*, %					.111
Acute symptomatic seizures	272	37.8	50	51.0	
Remote symptomatic unprovoked seizures	124	17.2	15	15.3	
Progressive CNS disorders	95	13.2	11	11.2	
Epilepsy without additional triggers (with or without underlying electroclinical syndrome)	151	21.0	12	12.2	
Cryptogenic	77	10.7	10	10.2	
Presumed fatal etiologies, *n*, %^16^, ^17^	225	31.3	39	39.8	.090
Main presumed SE etiologies (non‐mutually exclusive), *n*, %					
Known epilepsy	175	24.3	17	17.4	
Brain tumors	92	12.8	11	11.2	
Remote ischemic stroke	83	11.5	6	6.1	
Intracranial hemorrhage	68	9.4	15	15.3	
Drug withdrawal	38	5.3	11	11.2	
Traumatic brain injury	33	4.6	8	8.2	
Infectious (meningo‐)encephalitis	31	4.3	9	9.2	
Neurodegenerative	33	4.6	4	4.1	
Acute ischemic stroke	30	4.2	4	4.1	
Metabolic/electrolyte derangement	25	3.5	3	3.1	
Autoimmune encephalitis	26	3.6	2	2.0	
Septic encephalopathy	10	1.4	1	1.0	
Intoxication	8	1.1	1	1.0	
Posterior reversible encephalopathy syndrome	6	.8	1	1.0	
Drug effects	1	.1	0	.0	
SE type, *n*, %					
SE with prominent motor symptoms	302	41.9	30	30.6	.032
SE with motor symptoms (convulsive)	204	28.3	21	21.4	
SE with motor symptoms (myoclonic)	98	13.6	9	9.2	
SE without prominent motor symptoms	418	58.1	68	69.4	.032
NCSE with coma	143	19.9	46	46.9	**<.001**
NCSE without coma	275	38.2	22	22.4	.**002**
Generalized NCSE without coma (i.e., atypical absence status)	6	.8	0	.0	
Focal NCSE without coma (conscious)	50	6.9	2	2.0	
Focal NCSE without coma (with altered consciousness)	219	30.4	20	20.4	.044
Illness severity, median, IQR					
STESS	3	2–4	3	2–5	.**001**
CCI	4	2–6	3	2–5	.172
Initial level of consciousness and airway management, *n*, %					
Unconscious at SE onset	228	31.7	61	62.2	**<.001**
Intubation at SE onset	66	9.2	31	31.6	**<.001**

*Note*: Bold font indicates statistical significance.

Abbreviations: CCI, Charlson Comorbidity Index (range = 0–37)^20^; CNS, central nervous system; ILAE, international league against epilepsy^12^, ^15^; IQR, interquartile range; NCSE, nonconvulsive SE; RTI, respiratory tract infection; SE, status epilepticus; STESS, Status Epilepticus Severity Score (range = 0–6).^18^, ^19^

^a^ Level of significance set at *p* ≤ .002 after Bonferroni correction for multiple comparisons.

**TABLE 3:** Uni‐ and multivariable logistic regression analyses to identify associations at SE onset with the emergence of respiratory tract infection.

Clinical variables at SE onset	Univariable model				
	OR		95% CI	*p*	
NCSE with coma	3.57		2.31–5.53	**<.001**	
STESS	1.25		1.10–1.43	.**001**	
Unconscious at SE onset	3.56		2.30–5.51	**<.001**	
Intubation at SE onset	4.58		2.79–7.52	**<.001**	

Clinical variables at SE onset	Multivariable model				
	OR	95% CI	*p*	Hosmer–Lemeshow χ^2^	*p* ^a^
NCSE with coma	2.47	1.29–4.74	.**007**	7.90	.245
STESS	.86	.71–1.03	.099		
Unconscious at SE onset	2.23	1.24–4.01	.**008**		
Intubation at SE onset	2.67	1.54–4.64	.**001**		

Clinical variables at SE onset	Multivariable forward and backward selection (results equal for both models)				
	OR	95% CI	*p*	Hosmer–Lemeshow χ^2^	*p* ^a^
NCSE with coma	1.85	1.06–3.20	.**029**	3.76	.440
STESS	Not selected				
Unconscious at SE onset	1.93	1.10–3.38	.**022**		
Intubation at SE onset	2.61	1.51–4.53	.**001**		

Clinical variables at SE onset	Multivariable stepwise model selection by Akaike information criterion				
	OR	95% CI	*p*	Hosmer–Lemeshow χ^2^	*p* ^a^
NCSE with coma	2.47	1.29–4.74	.**007**	7.90	.245
STESS	.85	.71–1.03	.099		
Unconscious at SE onset	2.23	1.24–4.01	.**008**		
Intubation at SE onset	2.67	1.54–4.64	.**001**		

*Note*: Bold font indicates statistical significance.

Abbreviations: CI, confidence interval; NCSE, nonconvulsive SE; OR, odds ratio; SE, status epilepticus; STESS, Status Epilepticus Severity Score (range = 0–6).^18^, ^19^

^a^ Hosmer–Lemeshow goodness of fit tests for all models revealed insignificant *p*‐values, indicating adequate model fit for all models.

### 3.2 Secondary outcomes

Univariable comparisons of treatment characteristics and secondary outcomes between patients with and without respiratory tract infections are presented in Table 4.

**TABLE 4:** Univariable comparisons of secondary outcomes between patients with and without RTIs (*N* = 818).

Secondary outcomes	Patients without RTI during SE, *n* = 720		Patients with RTI during SE, *n* = 98		*p* ^a^
In‐hospital stay, days, median, IQR	12	5–22	21	11–30	**<.001**
SE duration, days, median, IQR	1	.5–1	2	.5–10	**<.001**
Persistent seizure termination, *n*, %	689	95.7	89	90.8	.045
Survival with return to premorbid neurologic function at discharge, *n*, %	318	44.2	20	20.4	**<.001**
In‐hospital death, *n*, %	57	7.9	21	21.4	**<.001**

*Note*: Bold font indicates statistical significance.

Abbreviations: IQR, interquartile range; RTI, respiratory tract infection; SE, status epilepticus.

^a^ Level of significance set at *p* ≤ .002 after Bonferroni correction for multiple comparisons.

Patients with SE and RTI had longer SE durations than patients with SE without RTI: median = 2 days (IQR = .5–10) versus 1 day (IQR = .5–1). Patients with SE and RTI also had longer hospital lengths of stay than patients with SE without RTI: median = 21 days (IQR = 11–30) versus 12 days (IQR = 5–22). Furthermore, patients with SE and RTI had higher in‐hospital mortality and lower rates of neurological recovery than patients with SE without RTI: mortality = 21.4% (21/98) versus 7.9% (57/720), return to premorbid neurological function at hospital discharge 20.4% (20/98) versus 44.2% (318/720). Linear and logistic regression analyses to identify associations between RTI and secondary outcomes are presented in Table 5. After adjusting for established outcome predictors (Table 5, median columns) including age, potentially fatal etiology, SE severity as quantified by the STESS, and the use of continuous anesthetics, RTI remained significantly associated with increased SE duration (regression coefficient = 8.44, 95% confidence interval [CI] = 5.31–11.56), a decreased likelihood of returning to premorbid neurological function (OR = .46, 95% CI = .26–.81), and increased in‐hospital death (OR = 2.12, 95% CI = 1.13–4.00). Of the 142 patients with infections during SE, 91.5% (130/142) of patients received antibiotics within a median of 0 days (IQR = 0–0) following the diagnosis of infection. The median duration of therapy was 7 days (IQR = 6–11). Patients with SE and RTI had higher peak levels of inflammatory blood‐based biomarkers than patients with SE without RTI (median peak CRP of 81 mg/L [IQR = 37–124] vs. 23 [IQR = 5–70], *p* < .001; peak leukocyte count of 11 × 10^9^/L [IQR = 9–15] vs. 10 × 10^9^/L [IQR = 8–13], *p* = .002). Additional multivariable models were performed including maximal serum levels of CRP and leukocytes (Table 5, right columns). These analyses revealed a slightly decreased level of significance for the associations between RTI and SE duration, survival with return to premorbid neurologic function, and in‐hospital death, suggesting a mediating effect on outcome by additional systemic inflammation emerging with RTI.

**TABLE 5:** Linear and logistic regression analyses to identify associations between RTI and secondary outcomes.

OR for secondary outcomes with RTI	Unadjusted			Adjusted for established predictors of outcome of SE^32^, ^33^			Adjusted for predictors of outcome of SE^32^, ^33^ and maximal CRP serum levels and leukocyte counts		
Secondary outcomes	Regression coefficient	95% CI	*p*	Regression coefficient	95% CI	*p* ^a^	Regression coefficient	95% CI	*p* ^a^
In‐hospital stay (for every additional day)	6.04	1.20–10.9	.**015**	.42	−4.40 to 5.24	.864	−.57	−5.44 to 4.3	.820
SE duration (for every additional day)	9.13	6.1–12.14	**<.001**	8.44	5.31–11.56	**<.001**	8.06	4.9–11.23	**<.001**

	OR	95% CI	*p*	OR	95% CI	*p* ^a^ ^,^ ^b^	OR	95% CI	*p* ^a^ ^,^ ^b^
Persistent seizure termination	.45	.21–.97	.**040**	.52	.22–1.23	.138	.49	.2–1.18	.113
Survival with return to premorbid neurologic function	.32	.19–.54	**<.001**	.46	.26–.81	.**007**	.54	.3–.98	.**041**
In‐hospital death	3.17	1.82–5.52	**<.001**	2.12	1.13–4.00	.**019**	1.98	1.05–3.72	.**034**

*Note*: Bold font indicates statistical significance.

Abbreviations: CI, confidence interval; CRP, C‐reactive protein levels (in mg/L); OR, odds ratio; RTI, respiratory tract infection; SE, status epilepticus.

^a^ Hosmer–Lemeshow goodness of fit tests for all models revealed insignificant *p*‐values, indicating adequate model fit.

^b^ Adjusted for established predictors of outcome of SE were age, potentially fatal etiology, Status Epilepticus Severity Score (including type of SE, level of consciousness at SE onset, and seizure history), and administered anesthetic drugs.

Sensitivity analysis assessing associations between RTI and secondary outcomes after exclusion of the subgroup of patients with VAP during SE revealed similar associations with secondary outcomes compared to RTI including VAP except for the loss of significance with in‐hospital death (Table S1).

## 4 DISCUSSION

This 20‐year longitudinal observational study conducted at a Swiss academic tertiary care center underscores the incidence and prognostic burden of RTIs in adult patients with SE. Among 818 patients, RTIs occurred in more than every eighth patient (12%) and more than every third patient (34.3%) requiring mechanical ventilation, with a median onset of 1 day following SE presentation. The observed overall incidence of RTI of 12% is slightly lower than the 16%–29% reported in earlier cohorts, likely reflecting the inclusion of a broader population of SE patients beyond those strictly requiring intensive care.^6^, ^25^ However, the median onset of RTI within 24 h aligns with previous research suggesting that infections in SE are early onset rather than late stage sequelae.^6^, ^8^, ^34^

RTIs are associated with specific clinical features at SE onset, namely NCSE with coma, unconsciousness, and the requirement for intubation early in the course of SE. The association between NCSE with coma and RTI development is consistent with the hypothesis that impaired consciousness leads to a loss of protective airway reflexes. This is consistent with findings in other neurocritical populations, such as stroke or traumatic brain injury, where “stroke‐associated pneumonia” is a well‐recognized driver of poor outcomes due to similar mechanisms of immunomodulation and aspiration risk.^35^

Even after adjusting for established confounders such as age, fatal etiologies, and the use of anesthetic agents, the presence of an RTI remained associated with a prolonged duration of SE, a doubled risk of in‐hospital mortality, and a significantly reduced likelihood of returning to premorbid functional baselines. These associations remained similar after exclusion of patients with VAP except the loss of significant increased in‐hospital mortality, indicating that with later emergence of RTI during SE its effect on mortality may increase. However, VAP may also reflect a more complicated and less controlled course of SE.

In addition, our study cannot answer whether increased mortality is directly related to RTI or to upregulated cytokines via increased systemic inflammation promoting ictal cerebral activity. However, the observation that RTI occurred more frequently in patients with longer duration of SE is consistent with the hypothesis that upregulated cytokines via systemic inflammation could promote cerebral ictal activity.^36^ In addition, maximal markers of systemic inflammation increased with the emergence of RTI, and multivariable models performed including maximal serum levels of CRP and leukocytes revealed a slightly decreased level of significance for the associations between RTI and SE duration, survival with return to premorbid neurologic function, and in‐hospital death, suggesting a mediating effect on outcome by additional systemic inflammation emerging with RTI. These results are corroborated by external data, including a recent 2024 study of 252 ICU patients with SE at a Belgian tertiary care center.^37^ That report identified infectious and respiratory complications as the most frequent adverse events and revealed their association with increased mortality.^37^ Earlier data from a Finnish academic center from 2006 involving 161 adult ICU patients with SE revealed a 42% infection rate.^8^ These patients required triple the ICU and hospital stay and nearly four times the nursing resources compared to those without infections. Our findings are consistent with a plausible mechanistically grounded rationale for evaluating the clinical utility of targeted antimicrobial prophylaxis in high‐risk patient subgroups. Our results demonstrate that RTI is not merely a marker of SE severity but has an association with seizure activity and treatment refractoriness. The regression analysis showed that RTI was associated with 8.44 more days of SE duration. This relationship may be bidirectional; SE and its treatment increase infection risk, and the systemic inflammatory response triggered by pneumonia can lower the seizure threshold and exacerbate neuronal hyperexcitability. This is supported by recent meta‐analyses in neurocritical care that suggest prophylactic antibiotics can reduce VAP and ICU length of stay, highlighting the clinical benefit of early infection prevention.^3^, ^4^ The choice of antimicrobial agent requires a cautious, evidence‐based approach to avoid iatrogenic exacerbation of SE due to the proconvulsant potential of several antibiotic classes.^38^, ^39^, ^40^ In a population already suffering from SE, these agents could counteract the benefits of infection prevention by promoting treatment refractoriness. Conversely, macrolides or certain aminoglycosides may carry a lower risk of neurotoxicity but might not provide the necessary spectrum of coverage.^41^ Third generation cephalosporins might be the antibiotics of choice given the data on antibiotic prophylaxis in other brain injuries.^3^, ^4^

Given that more than every second patient with NCSE, coma, and intubation developed an RTI, this subpopulation may derive the greatest benefit from early antimicrobial intervention. Consistent with the principles of antimicrobial stewardship, the goal would not be universal administration, but rather a targeted approach for those whose respiratory risk profile poses the greatest threat to their neurological prognosis.

### 4.1 Strengths and limitations

A primary advantage of this research lies in the large and representative sample of adult patients with SE, classified strictly according to ILAE criteria and subjected to rigorous adjustment for confounding variables, as well as the adherence to the STROBE guidelines. Given that the clinical profiles within our cohort correspond significantly with existing SE literature, our results likely maintain high generalizability across comparable clinical environments. Expert oversight by epileptologists and neurocritical care specialists ensured clinical management met international standards.

Despite these findings, several limitations remain. The single‐center, observational design may limit generalizability. Additionally, SE duration, particularly in NCSE cases, might be underestimated due to the difficulty of timing unwitnessed onsets.^42^ Finally, the retrospective nature of the study precluded an accurate count of superrefractory SE, as we could not reliably distinguish anesthesia escalation for persistent SE from adjustments made for side effects or recurrence prevention. In addition, we acknowledge that our study is not designed to prove causality and can only point to associations and inform hypotheses. We also acknowledge that although adjusting for peak overall inflammation levels accounts for some effects of concurrent non‐RTI infections during SE, further specific adjustments for these infections were not performed. This decision was made due to the high diversity in types and severity of non‐RTI infections, combined with the low sample size for each specific infection type.

Importantly, the observational design of the included studies precludes conclusions regarding the direction of the observed associations. Patients with more severe SE may be inherently more susceptible to developing respiratory tract infections, respiratory tract infections may contribute to a more severe clinical course, or both mechanisms may coexist.

Lastly, although at our institution the diagnostic and treatment procedures for RTI are standardized as outlined, another limitation may come with challenges in consistently classifying RTI retrospectively.

## 5 CONCLUSIONS

Our study found a high incidence of RTIs emerging during SE, affecting more than one third of mechanically ventilated patients and half of those intubated for NCSE with coma. Linked to coma and early intubation, these infections are associated with doubled mortality rates and significantly hinder neurological recovery. Prospective studies are needed to clarify the temporal and causal relationships between RTIs and SE severity and to determine whether preventive or therapeutic strategies targeting RTIs improve clinical outcomes.

## AUTHOR CONTRIBUTIONS

Raoul Sutter and Sebastian Berger contributed to the study conception and design. Sarah Tschudin‐Sutter and Raoul Sutter completed the statistical analysis. Sebastian Berger, Sarah Tschudin‐Sutter, and Raoul Sutter devised the original draft of the manuscript. All authors provided substantial editing of the manuscript. All authors read and approved the final manuscript.

## CONFLICT OF INTEREST STATEMENT

The authors declare that they have no conflicts of interest relevant to this study.

## ETHICAL APPROVAL STATEMENT

We confirm that we have read the Journal's position on issues involved in ethical publication and affirm that this report is consistent with those guidelines. This study was conducted in accordance with the ethical standards of the institutional and national research committee and with the principles of the Declaration of Helsinki and its later amendments. The study protocol was approved by the ethics committee of Northwestern and Central Switzerland (EKNZ 2019‐00693).

## CONSENT FOR PUBLICATION

All participants provided consent for the publication of anonymized data included in this article.

## Supporting information


**TABLE S1.** Linear and logistic regression analyses to identify associations between respiratory tract infection and secondary outcomes after exclusion of patients with ventilator‐associated pneumonia (*n* = 21; sensitivity analyses).

## Data Availability

The datasets generated and/or analyzed during the current study are available from the corresponding author on reasonable request.
