# Supplementary material for: Incidence and impact of respiratory tract infections on outcomes in patients with status epilepticus: A 20‐year longitudinal observation
Source: Epilepsia. 2026 Sep 17;67(11):5875–88. doi: 10.1002/epi.70458 (PMC13768911; doi:10.1002/epi.70458)
Supplement: Supplementary file 1 [file EPI-67-5875-s001.docx]

**Supplemental Table 1:** Linear and logistic regression analyses to identify associations between RTI and secondary outcomes after exclusion of patients with VAP (n=21; sensitivity analyses)

| **OR for secondary outcomes with RTI excluding VAP** | **Unadjusted** | | | | **Adjusted for established predictors of outcome of SE (27,28)** | | | |
| --- | --- | --- | --- | --- | --- | --- | --- | --- |
| **Secondary outcomes** | **regr. coeff.** | **95% CI** | **p-value** | **regr, coeff.** | | **95% CI** | **p-value^#^** |  |
| In-hospital stay  (for every additional day) | 4.06 | -1.35-9.5 | 0.141 | -0.45 | | -5.77-4.87 | 0.868 |  |
| SE duration  (for every additional day) | 8.47 | 5.11-11.8 | **<0.001** | 8.00 | | 4.56-11.43 | **<0.001** |  |
|  | **OR** | **95% CI** | **p-value** | **OR** | | **95% CI** | **p-value^#^*** |  |
| Persistent seizure termination | 0.45 | 0.19-1.06 | 0.068 | 0.56 | | 0.22-1.42 | 0.221 |  |
| Survival with return to premorbid neurologic function | 0.36 | 0.22-0.67 | **0.001** | 0.52 | | 0.28-0.96 | **0.036** |  |
| In-hospital death | 2.59 | 1.36-4.90 | **0.004** | 1.76 | | 0.86-3.61 | 0.120 |  |

RTI = respiratory tract infection; VAP = ventilator associated pneumonia; OR = odds ratio; CI = confidence interval

*Adjusted for established predictors of outcome of SE were age, potentially fatal etiology, the STESS (including type of SE, level of consciousness at SE onset, and seizure history), and administered anesthetic drugs

^#^Hosmer Lemeshow goodness of fit tests for all models revealed insignificant p-values indicating adequate model fit

**Bold font** indicates statistical significance.
